# Outcomes of Diabetic and Nondiabetic Patients Undergoing General and Vascular Surgery

**DOI:** 10.1155/2013/963930

**Published:** 2013-12-26

**Authors:** Stephen Serio, John M. Clements, Dawn Grauf, Aziz M. Merchant

**Affiliations:** ^1^Department of Surgery, Covenant Healthcare, Central Michigan University College of Medicine, Saginaw, MI 48603, USA; ^2^Department of Research, Covenant Healthcare, Central Michigan University College of Medicine, Saginaw, MI 48603, USA; ^3^Department of Quality, Covenant Healthcare, Saginaw, MI 48603, USA

## Abstract

*Aims*. Preoperative diabetic and glycemic screening may or may not be cost effective. Although hyperglycemia is known to compromise surgical outcomes, the effect of a diabetic diagnosis on outcomes is poorly known. We examine the effect of diabetes on outcomes for general and vascular surgery patients. *Methods*. Data were collected from the Michigan Surgical Quality Collaborative for general or vascular surgery patients who had diabetes. Primary and secondary outcomes were 30-day mortality and 30-day overall morbidity, respectively. Binary logistic regression analysis was used to identify risk factors. *Results*. We identified 177,430 (89.9%) general surgery and 34,006 (16.1%) vascular surgery patients. Insulin and noninsulin diabetics accounted for 7.1% and 9.8%, respectively. Insulin and noninsulin dependent diabetics were not at increased risk for mortality. Diabetics are at a slight increased odds than non-diabetics for overall morbidity, and insulin dependent diabetics more so than non-insulin dependent. Ventilator dependence, 10% weight loss, emergent case, and ASA class were most predictive. *Conclusions*. Diabetics were not at increased risk for postoperative mortality. Insulin-dependent diabetics undergoing general or vascular surgery were at increased risk of overall 30-day morbidity. These data provide insight towards mitigating poor surgical outcomes in diabetic patients and the cost effectiveness of preoperative diabetic screening.

## 1. Introduction

The national diabetes epidemic continues to expand, with about 1.6 million new cases each year and an overall prevalence of 23.6 million people. Additional 57 million American adults are at high risk for type 2 diabetes mellitus (T2DM) [1]. The annual diagnosed diabetes incidence is projected to almost double from 8 cases per 1,000 in 2008 to about 15 in 1,000 by 2050, with a potential total prevalence projected to increase one-fifth of the US population by 2050 [2]. Currently, 10% of Americans have T2DM, and 20% to 25% are considered prediabetic with impaired glucose tolerance and elevated fasting glucose measurements [3]. The long-term complications of uncontrolled hyperglycemia in diabetics include neuropathy, nephropathy, retinopathy, and other chronic issues. The in-hospital morbidity of diabetics and new-onset hyperglycemics is well documented. Umpierrez et al. showed that for inpatients with new hyperglycemia or known diabetes there was a higher in-hospital mortality, increased length of stay, increased intensive care unit (ICU) stay, increased transitional or nursing home care, a higher rate of infections, and more neurologic events compared to a normoglycemic control group [4]. In addition, diabetes and inpatient hyperglycemia increase mortality for patient with an acute myocardial infarction [5, 6]. The role of diabetes in outcomes of the critically ill, intensive care unit patient continues to be debated [7–10].

Diabetics are increasingly undergoing elective and emergent surgery; however, whether the presence of diabetes compromises surgical outcomes remains unclear. It is accepted that diabetics have an increased risk of cardiovascular disease compared to nondiabetics [11]. King et al. have shown that diabetics undergoing general and vascular surgery with postoperative hyperglycemia exceeding 150 mg/dL had increased rates of infection [12]. Moreover, Bower et al., in a study of prospective patient registries, showed increased morbidity in known diabetic patients with and without malignancy and that diabetes was related to degree of postoperative complications in a quantitative fashion [13]. In addition, the detriment of uncontrolled hyperglycemia in surgical patients has been investigated [14, 15].

In specific surgical populations, particularly cardiac and vascular patients, the negative effect of diabetes on postoperative outcomes is clear. Patients undergoing surgery are subject to altered carbohydrate metabolism, increased production of glucose, and increased resistance to insulin, leading to a stress-induced hyperglycemia. In cardiac surgery patients, maintenance of perioperative blood glucose levels between 125 and 200 mg dL^−1^ resulted in fewer episodes of atrial fibrillation and recurrent ischemia, as well as a shorter length of stay (LOS) [16, 17]. Similarly, in the vascular population suboptimal Hgb A1c levels in diabetics and non-diabetics predicted poor postoperative outcomes [18]. In other surgical specialties, the association is mild. Diabetics undergoing lumbar spine surgery fared worse than non-diabetics for spine-specific outcomes postoperatively [19]. However, the neurosurgical literature reveals only modest benefits linked to strict control of glycemic indexes during acute periods of brain ischemia [20] and in specific surgical situations such as spine [21] and tumor surgery [22]. A diagnosis of diabetes has not been shown to be an independent risk factor for poor outcomes in general surgery.

Studies investigating overall surgical outcomes of patients with a known diagnosis of diabetes, irrespective of perioperative hyperglycemia, are sparse, and most of these are fraught with small sample sizes, are specific to only one surgical specialty, are or guided towards one specific outcome measure (e.g., surgical site infection). We asked the question of whether a diagnosis of insulin dependent or non-insulin dependent diabetics impacted overall postoperative morbidity and mortality in a large administrative general and vascular surgical population. We sought to answer this question through query of a comprehensive statewide surgical outcomes database.

## 2. Materials and Methods

### 2.1. Database

This study was categorized as exempt from review at both Central Michigan University (CMU) College of Medicine and Covenant Healthcare. We used data from the Michigan Surgical Quality Collaborative (MSQC) for this study. Briefly, The MSQC is a statewide quality improvement effort representing 52 member hospitals in the state of Michigan. It is modeled after the American College of Surgeons' National Surgical Quality Improvement Program. Over 200 variables are collected about surgical interventions using reliable and validated systematic sampling techniques to provide a representative sample of cases from each participating hospital. The MSQC prospectively collects data on (1) thirty-day morbidity, (2) preoperative laboratory values, (3) readmissions and unplanned return to the operating room, (4) preoperative risk factors, (5) postoperative laboratory values and discharge variables, and (6) 30-day postoperative mortality. At larger hospitals, cases are selected by systematic sampling. The data is risk-adjusted by the MSQC team regularly. In addition, quarterly and twice-a year reports are generated for participating hospitals to aid in quality improvement. Data is collected on over 3000 Current Procedural Terminology (CPT) codes.

### 2.2. Patients

We included subjects from the MSQC database from the years 2007 through 2011 who had general or vascular surgery and diabetes status recorded and were over 18 years of age.

### 2.3. Variables

The primary outcome variable was 30-Day Mortality. In addition, we created a secondary outcome variable, Any Major Morbidity, from six variables in the MSQC database detailing postoperative occurrences: Wound, Respiratory, Urinary Tract, Central Nervous System, Cardiac, and Other Occurrences. Subjects experiencing one or more of these post-operative occurrences were classified as having Any Major Morbidity.

We also used a range of variables from the MSQC database to model our outcome variables. Preoperative Risk variables included history of Diabetes, Smoking, Dependence on Ventilator within 48 hours, COPD, Dialysis, Steroid Use, ≥10% Weight Loss within Six Months, and Sepsis within 48 hours. Subjects were classified as having a Cardiac Preoperative Risk if they had any of the following: congestive heart failure (CHF) within 30 days, history of myocardial infarction (MI), previous percutaneous coronary intervention (PCI) or percutaneous transluminal coronary angioplasty (PTCA), previous cardiac surgery, history of angina within 30 days, or on hypertensive medications. Finally, we included OR Time, Wound classification, ASA Class, whether cases were Emergent, and type of surgery: general or vascular.

### 2.4. Specific Definitions

CHF within 30 days is defined by the MSQC as CHF, congestive heart failure, or pulmonary edema. A history of MI within past 6 months is defined as a history of a non-Q wave or a Q wave myocardial infarct in the six months prior to surgery. A history of previous PCI/PTCA includes balloon dilatation and stent placement; however, it does not include valvuloplasty. Previous cardiac surgery includes procedures classified as “off-pump” repair or utilizing cardiopulmonary bypass, valve replacement or repair, great thoracic vessel repair, cardiac transplant, repair of atrial or ventricular septal defects, left ventricular aneurysmectomy, insertion of left ventricular assist devices, and so forth; however, it does not include insertions of pacemakers or automatic implantable cardioverter defibrillators. Hypertension requiring medication is defined as a persistent elevation of systolic pressure over 140 mmHg or a diastolic pressure over 90 mmHg or requires an antihypertensive treatment, at the time the patient is being considered for surgery. Emergency cases are defined as being performed as soon as possible and no later than 12 hours after hospital admission or onset of appropriate symptoms. Steroid use is defined as the regular administration of oral or intravenous corticosteroids 30 days prior to surgery for a chronic condition and does not include topical, rectal, or inhalation corticosteroids or a course of short course steroids (10 days or less).

### 2.5. Statistical Analysis

A summary of descriptive statistics for all variables is presented in Table 1. We conducted binary logistic regression models for 30-Day Mortality (Table 2) for the entire sample (Model 1), a subgroup of diabetics with general surgery (Model 2), and a subgroup of diabetics with vascular surgery (Model 3). Similarly, we conducted binary logistic regression models for Any Morbidity (Table 3) for the entire sample (Model 4), a sub-group of diabetics with general surgery (Model 5), and a subgroup of diabetics with vascular surgery (Model 6). All models were saturated models, that is, using all independent variables within each dependent variable (mortality and morbidity) to see which independent variables had the highest contribution to the desired outcome. All analyses were done using Statistical Product and Service Solutions (SPSS) Version 20 (IBM, Armonk, NY).

## 3. Results

### 3.1. Sample Description

A complete demographic and variable summary of our sample is presented in Table 1. Of 211,436 subjects, there were 177,430 (83.9%) general surgeries and 34,006 (16.1%) vascular surgeries. Males comprised 42.6% of subjects, and the average age was 56.5 ± 17.1. There were 175,639 (83.1%) nondiabetics, 15,050 (7.1%) insulin dependent diabetics, and 20,747 (9.8%) diabetics who were dependent on oral medications. Overall thirty-day mortality was two percent (4,083, 1.9%) and 13.0% (27,533) of cases were observed to have any type of major morbidity [23].

In addition, 24% of subjects were smokers with the past 12 months, 6% had chronic obstructive pulmonary disease (COPD), and 50.8% had some level of cardiac risk. 84% of patients were either ASA class 2 or 3, and 52.7% of wounds were classified as clean. About 1 out of 8 cases were Emergent.

Our goal was to determine if specific patient characteristics influenced mortality and morbidity and so we began by running regression analyses on these variables. Interestingly, our preliminary analyses indicated that smoking is not associated with an increased risk of mortality. While this is unexpected based on most other studies that associate smoking with increased mortality, in our sample, there is a negative correlation between smoking and diabetes. In addition, smokers were more likely to have anesthesia types other than general anesthesia. In both cases (diabetes and anesthesia other than general) the risk of mortality is decreased and the association of smoking with these characteristics likely impacts the influence of smoking. Due to the high multicollinearity of smoking with several other characteristics, we removed smoking as a variable from the regression analyses reported in Section 3.2.

### 3.2. Regression Analyses—30-Day Mortality

We regressed the 30-Day Mortality variable using three binary logistic regression models (Table 2) to determine the ability of different variables to predict mortality in diabetic subgroups of general and vascular surgery patients. Model 1 uses the entire sample. In this case, those subjects who died within 30 days were less likely to be oral (OR = 0.78, 95% CI 0.70–0.86) or insulin (OR = 0.83, 95% CI 0.75–0.92) dependent diabetics than not diabetic. In our sample, diabetes does not increase the risk of 30-Day Mortality. Vascular surgery (compared to general surgery) is a significant predictor of mortality (OR = 1.57, 95% CI 1.42–1.72) for the entire group.

Due to our interest in the diabetic population, we conducted further subgroup analyses for 30-Day Mortality using only diabetic patients. Model 2 (Table 2) is a subgroup analysis of diabetic, general surgery subjects. In this case, there is no difference between oral and insulin dependent diabetics in survival. Model 3 (Table 2) is a subgroup analysis of diabetic, vascular surgery subjects. As with the general surgery subgroup, there is no difference between oral and insulin dependent vascular surgery, diabetics in survival.

Moving from the consideration of diabetes as a variable, we also included our other variables in the analyses. In general and as we would expect, ventilator dependence, COPD, cardiac risk factors, current dialysis, steroid use, weight loss, sepsis, emergent cases, increased ASA class, and any wound classification increase the risk of 30-Day Mortality.

In summary, our models for 30-Day Mortality show that for our entire sample, the presence of diabetes does increase the risk of 30-Day Mortality. We provide possible reasons for this observation in our discussion. Vascular surgery is predictive of mortality. There do not appear be significant differences in variables that contribute to mortality, when comparing models for diabetic subgroups of general and vascular surgery and, as expected, ventilator dependence, COPD, current dialysis, weight loss, sepsis, emergent cases, increased ASA and any wound classification increase the risk of 30 day mortality in diabetic patients.

### 3.3. Regression Analyses—Any Morbidity

We also regressed the Any Morbidity variable using three binary logistic regression models (Table 3) to determine the ability of different variables to predict any type of morbidity in different subgroups of surgery patients. Model 4 uses the entire sample. Subjects with any type of morbidity were more likely to be insulin dependent (OR = 1.11, 95% CI 1.06–1.16) diabetics than oral dependent diabetics, or not diabetic. Vascular surgery (compared to general surgery) is also a significant predictor of morbidity (OR = 1.69, 95% CI 1.62–1.75).

Model 5 (Table 3) is a subgroup analysis of diabetic, general surgery subjects. In this case, insulin dependent diabetics are more likely to have any morbidity (OR = 1.20, 95% CI 1.12–1.29) when compared to diabetics who are dependent on oral medications. Model 6 (Table 3) is a subgroup analysis of diabetic, vascular surgery subjects. Again, we found that insulin dependent diabetics are more likely to have any morbidity (OR = 1.24, 95% CI 1.12–1.38) when compared to oral medication dependent.

Again, considering other variables across all models for Any Morbidity, we found that in general, ventilator dependence, COPD, cardiac risk factors, current dialysis, steroid use, weight loss, sepsis, emergent cases, increased ASA class, and any wound classification increase the risk of any morbidity.

In summary, models for Any Morbidity show that for our entire sample, insulin dependent diabetics have an increased risk for any morbidity (OR = 1.11, 95% CI 1.06–1.16) when compared to non-diabetics. This increased risk is also present in subgroup analyses comparing insulin dependent diabetics to oral medication dependent diabetics for general (OR = 1.20, 95% CI 1.12–1.29) and vascular (OR = 1.24, 95% CI 1.12–1.38) surgeries. This finding was not evident for the 30-Day Mortality outcome. Again, vascular surgery is predictive of morbidity. As with the mortality analyses, there do not appear be significant differences in variables that contribute to morbidity, when comparing models for diabetic subgroups of general and vascular surgical patients. As expected, ventilator dependence, COPD, current dialysis, weight loss, sepsis, emergent cases, increased ASA and any wound classification increase the risk of any morbidity in diabetic patients.

## 4. Discussion

Our objective was to understand the postoperative risk for morbidity and mortality in diabetics versus non-diabetic patients undergoing general or vascular surgery. We found that, with regard to mortality, the presence of diabetes is not predictive of mortality; however, vascular surgery itself is predictive of mortality. There are no significant differences in variables that contribute to mortality, when comparing diabetic and non-diabetic subgroups for both general and vascular surgery. In our morbidity analysis, insulin dependent diabetics have an increased risk for any morbidity compared to non-diabetics or non-insulin (oral) dependent diabetics in the general and vascular surgery subgroups. Our multivariate analyses confirm findings in the literature of risk factors in general surgery that contribute to morbidity and mortality, such as ventilator dependence, COPD, cardiac risk factors, dialysis, steroid use, significant weight loss, sepsis, emergent cases, and increasing ASA class.

Interestingly, notable differences were detected when analyzing separate groups of diabetics and non-diabetics undergoing general surgery. For instance, male non-diabetics undergoing general surgery have an increased risk for any morbidity as compared male diabetics. It is possible that higher risk male diabetics were more likely denied elective surgical intervention, as opposed to higher risk non-diabetic males, resulting in our finding. As in the mortality analysis, there do not appear be significant differences in variables that contribute to morbidity for diabetics and non-diabetics specifically undergoing vascular surgery.

This is the first study in the literature of overall post-operative outcomes (morbidity and mortality) of diabetics undergoing general surgery with such a large population of patients. Schlussel et al. examined 55,000 diabetic patients in the Veterans Heath Administration National Surgical Quality Improvement Program database and analyzed the effect of different levels of preoperative hyperglycemia on the rate of wound infection [10]. However, in their cohort, there was no comparison of diabetics to a non-diabetic control group, as that was not the purpose of their study. Within their study, they showed that higher preoperative serum glucose levels correlated with worse wound-related outcomes; however, preoperative HbA1c (long-term glucose control in diabetics) had no association with infection rates. In a study of preoperative HbA1c levels, O'Sullivan found that, after multivariate analysis, elevated HbA1c levels were predictive of morbidity in non-diabetic patients, but not in diabetics, undergoing vascular surgery [24]. There was no effect on mortality, consistent with our findings. The O'Sullivan study does highlight the disturbing problem of undiagnosed diabetics undergoing surgery without proper control of their glycemia.

The lack of correlation between diabetic diagnosis and mortality in general surgery in our study is not unique in the literature, where a clear consensus on the issue cannot be found. Guckelberger et al. showed in a study of over 600 patients undergoing hepatic resection that multivariate analysis did not identify diabetes as an independent variable having an impact on mortality and that overall complications were equally frequent between diabetic and non-diabetic patients [25]. Diabetes was also shown not to be a statistically significant predictor of overall mortality or cardiac morbidity in patients undergoing open aortic aneurysm repair [26]. In a matched cohort retrospective review, diabetes did not increase the mortality rates of cardiac surgery, but increased the risk for renal and neurological complications, blood transfusion, reoperation, and length of ICU stay [27]. However, a smaller study than ours from Finland, with a long 7-year followup, showed quite convincingly that short-term and long-term mortality was higher in diabetics versus non-diabetics undergoing similar noncardiac surgeries [28]. Juul et al. described a high mortality, due mainly to cardiovascular complications, in cohort of diabetics undergoing noncardiac surgery; however, there was no non-diabetic control group in the study [29].

Our study revealed that the presence of cardiac risk predicted overall morbidity in diabetic and non-diabetics undergoing general and vascular surgery equally. However, cardiac risk predicted mortality only in non-diabetic general and vascular surgery patients. Conversely, Hollenberg et al. showed that diabetes was one of five risk factors predicted of cardiac events in patients undergoing non-cardiac surgery [30]. One difference that may account for the results is that the latter study began from the premise of a population of patients that were already high risk for a cardiac event occurring and underwent non-cardiac surgery. In contrast, our study evaluated all patients from the perspective of being diabetic or not and assessing factors predictive of morbidity and mortality in diabetic versus non-diabetic, that is, a difference of study design to test a different hypothesis.

Most studies regarding surgical outcomes of diabetics involve studying the presence of perioperative hyperglycemia in diabetics or non-diabetics and its effect on morbidity and mortality. Currently, the literature regarding optimal target glucose ranges for the perioperative patient is unclear. The ADA recommends screening for diabetes preoperatively in select populations, especially in obese patients [31]. The United States Preventive Services Task Force (USPSTF) recommends screening only hypertensive adults for diabetes [32]. Some of the first studies to evaluate the benefits of tight glucose control were the Leuven study in 2001 and 2006, which sparked widespread interest in intensive insulin therapy in critically ill patients [33, 34]. Some researchers relate the benefits identified in this study to our current understanding of the bodies stress response. In the Leuven study, the majority of the critically ill patients studied had spent more than five days in the intensive care unit. The effects of an anabolic steroid, such as insulin, may be more beneficial as the response to the acute injury resolves. However, during the acute onset of stressful stimulus such as surgery or possibly traumatic events the effect of treatment with these hormones has not been fully explained and may be harmful [35].

Despite the fact, improvements in surgical outcome have been seen in patients with tight intraoperative glucose control. As previously mentioned, a growing body of evidence suggests that even small changes in glycemic ranges are associated with impaired outcomes. In one study, in patients undergoing cardiac surgery, a 20 mg/dL increase in mean intraoperative glucose was associated with increased risk of more than 30% of adverse outcome [36]. However, it is important to make the distinction that these findings cannot be generalized to general surgical patients. The broad array of patient demographics and options for surgical care encompass an extensive variety of settings, and as many operations performed by the modern general surgeon are now performed in the ambulatory setting, the assessment and management of the dysglycemia patient continue to be a looming challenge.

The MSQC is an excellent source of data for studying general and specific 30-day morbidity for various surgical procedures on a larger scale. The administrative database provides risk and reliability adjusted reports for over 52 participating hospitals in the state of Michigan. The other option to answer our question would have been to use the National Surgical Quality Improvement Program (NSQIP) database or the National Inpatient Sample (NIS) hospital discharge data. Our hospital currently does not participate in the NSQIP system. The NIS system, although widely available and currently the largest all-payer database of hospital discharge records, poses a number of limitations. First, inconsistencies across states and providers in the data element reporting may compromise data quality. Second, other data elements that would be needed to analyze risk factors for certain preoperative patient populations, such as race/ethnicity, detailed test results, whether a condition was present on admission, functional status, severity of illness, and behavioral risk factors, would be absent in many cases.

Our analysis of a large administrative database has certain limitations. First, collaboration in the MSQC is purely voluntary, and these hospitals may have different characteristics from those who have chosen not to participate. Therefore, our results may not be generalizable to the entire cohort of statewide hospitals. Moreover, Michigan hospital practices may not be representative of US hospitals, although this database includes academic and community as well as various sizes and geographic locations within the state. Secondly, data in the MSQC is collected retrospectively and analyzed by the end-user retrospectively and therefore will have the biases inherent to such a study design. Third, the present analysis does not address procedure specific outcomes for diabetes. For example, although a cholecystectomy may not have the level of morbidity or mortality as a pancreatic operation for a diabetic, the volume of cholecystectomies in the database outweighs that of complex pancreatic operations. Although cases are easily distinguished between vascular and nonvascular surgeries, we did not separate out or group the 3000 CPT codes to identify procedure specific risk.

## 5. Conclusion

In summary, we found that a diagnosis of diabetes (insulin or oral dependent) did not affect outcomes in general surgical patients. However, the presence of insulin-dependent diabetes was associated with increased morbidity in the vascular and general surgery subgroups greater than non-diabetics or non-insulin dependent diabetics. What is unclear from our study is the underlying mechanism for this difference. However, the reassuring fact that the presence of diabetes did not have an overt effect on mortality, along with morbidity risk of insulin-dependent diabetes, may greatly impact the surgeon-patient preoperative dialogue and our ability to appropriately risk-stratify and risk-optimize these patients.

## Figures and Tables

**Table 1:** Study variables and demographics.

	*N *	Percent
Dependent variables		
30-day mortality	4083	1.9%
Any Major Post-Op Morbidity	27533	13.0%
Independent variables		
Diabetes		
Not present	175639	83.1%
Oral	20747	9.8%
Insulin dependent	15050	7.1%
Anesthesia technique		
General	185868	87.9%
Epidural	978	0.5%
Spinal	5462	2.6%
Regional	1891	0.9%
Local	56	0.0%
Monitored anesthesia care	3002	1.4%
Other	13668	6.5%
None	511	0.2%
Surgical speciality		
General	177430	83.9%
Vascular	34006	16.1%
Gender: male	90088	42.6%
Smoking within 1 year	50541	23.9%
Ventilator within 48 hours	2057	1.0%
COPD	12770	6.0%
Cardiac risk	107498	50.8%
Dialysis	4984	2.4%
Steroid use	5825	2.8%
10% weight loss	3537	1.7%
Sepsis within 48 hours		
None	192081	90.8%
Sepsis	5833	2.8%
Septic shock	1827	0.9%
SIRS	11695	5.5%
Emergent cases	26543	12.6%
ASA class		
No ASA class assigned	2020	1.0%
No disturbance	16381	7.7%
Mild disturbance	96184	45.5%
Severe disturbance	81339	38.5%
Life threatening	14870	7.0%
Moribund	642	0.3%
Wound classification		
Clean	111430	52.7%
Clean/contaminated	68374	32.3%
Contaminated	17147	8.1%
Dirty/infected	14485	6.9%

COPD: chronic obstructive pulmonary disease, ASA: American Society of Anesthesiologists, and SIRS: systemic inflammatory response syndrome.

**Table 2:** Logistic regression models for predicting 30-day mortality.

Variable	Model 1—entire sample	Model 2—diabetics with general surgery subgroup	Model 3—diabetics with vascular surgery subgroup
	Model *R*-square		
	0.333	0.300	0.165
	Odds ratio (95% CI)	Odds ratio (95% CI)	Odds ratio (95% CI)
Diabetes			
Nondiabetic	Reference category		
Diabetic—oral	0.78 (0.70, 0.86)*	Reference category	Reference category
Diabetic—insulin	0.83 (0.75, 0.92)*	1.18 (1.00, 1.39)	1.06 (0.83, 1.35)
Anesthesia technique			
General	Reference category	Reference category	Reference category
Epidural	0.46 (0.24, 0.91)*	0.00 (0.00, *α*)	0.85 (0.34, 2.11)
Spinal	0.87 (0.68, 1.09)	0.25 (0.06, 1.01)	1.25 (0.85, 1.82)
Regional	0.48 (0.30, 0.76)*	1.08 (0.14, 8.16)	0.29 (0.11, 0.79)
Local	0.43 (0.19, 0.95)*	0.00 (0.00, *α*)	0.09 (0.003, 2.33)
Monitored anesthesia care	0.78 (0.64, 0.96)*	0.97 (0.62, 1.51)	0.96 (0.62, 1.49)
Other	1.06 (0.46, 2.44)	1.14 (0.15, 8.69)	0.00 (0.00, *α*)
None	3.60 (1.03, 12.6)*	0.00 (0.00, *α*)	0.00 (0.00, *α*)
Surgical specialty: vascular	1.57 (1.42, 1.72)*		
Male gender	0.99 (0.92, 1.06)	1.15 (0.98, 1.35)	0.92 (0.75, 1.16)
Ventilator dependent	2.19 (1.93, 2.49)*	2.13 (1.63, 2.79)*	3.54 (2.13, 5.88)*
COPD	1.61 (1.47, 1.76)*	1.54 (1.26, 1.88)*	1.54 (1.17, 2.02)*
Cardiac risk factors	1.57 (1.43, 1.72)*	1.18 (0.91, 1.152)	1.49 (0.87, 2.53)
On dialysis	1.39 (1.24, 1.56)*	1.85 (1.46, 2.35)*	1.88 (1.46, 2.42)*
Steroid use	1.31 (1.16, 1.48)*	1.29 (0.98, 1.70)	1.15 (0.73, 1.8)
10% weight loss	2.81 (2.45, 3.22)*	2.73 (1.98, 3.77)*	2.25 (1.24, 4.08)*
Sepsis			
None	Reference category	Reference category	Reference category
Sepsis	2.62 (2.32, 2.96)*	2.49 (1.92, 3.23)*	1.84 (1.26, 2.68)*
Septic shock	3.39 (2.93, 3.94)*	3.89 (2.85, 5.34)*	3.42 (1.91, 6.14)*
SIRS	2.30 (2.08, 2.55)*	2.13 (1.67, 2.73)*	1.80 (1.29, 2.53)*
Emergent case	2.33 (2.15, 2.53)*	2.09 (1.73, 2.53)*	2.35 (1.78, 3.11)*
ASA class			
No ASA class assigned	Reference category	0.00 (0.00, *α*)	Reference category
No disturbance	0. 44 (0.014, 0.14)*	0.00 (0.00, *α*)	0.00 (0.00, *α*)
Mild disturbance	0.20 (0.08, 0.49)*	0.06 (0.033, 0.107)*	0.21 (0.005, 8.64)*
Severe disturbance	1.53 (0.62, 3.78)	0.20 (0.13, 0.32)*	0.41 (0.11, 15.2)*
Life threatening	5.40 (2.19, 13.2)*	0.64 (0.42, 0.99)*	0.89 (0.024, 33.0)*
Moribund	13.6 (5.44, 33.9)*	Reference category**	5.37 (0.13, 217.5)*
Wound classification			
Clean	Reference category	Reference category	Reference category
Clean/contaminated	2.01 (1.82, 2.22)*	2.40 (1.87, 3.09)*	2.03 (1.39, 2.96)*
Contaminated	1.88 (1.66, 2.14)*	2.45 (1.80, 3.33)*	1.34 (0.89, 2.02)
Dirty/infected	1.78 (1.58, 1.99)*	2.37 (1.76, 3.18)*	0.99 (0.71, 1.40)

Values expressed as odds ratios (95% confidence intervals *x*, *y*). *Significance at *P* < 0.05. **Moribund category is used as the reference category as there were no cases designated as “not assigned” or “no disturbance” and odds ratios could not be calculated in reference to these categories. COPD: chronic obstructive pulmonary disease, ASA: American Society of Anesthesiologists, SIRS: systemic inflammatory response syndrome, and CI: confidence interval.

**Table 3:** Logistic regression models for predicting 30-day morbidity.

Variable	Model 4—entire sample	Model 5—diabetics with general surgery sub-group	Model 6—diabetics with vascular surgery sub-group
	Model *R*-square		
	0.225	0.204	0.111
	Odds ratio (95% CI)	Odds ratio (95% CI)	Odds ratio (95% CI)
Diabetes			
Nondiabetic	Reference category		
Diabetic—oral	0.96 (0.92, 1.01)	Reference category	Reference category
Diabetic—insulin	1.11 (1.06, 1.16)*	1.20 (1.12, 1.29)*	1.24 (1.12, 1.38)*
Anesthesia technique			
General	Reference category	Reference category	Reference category
Epidural	0.97 (0.80, 1.12)	1.11 (0.51, 2.36)	1.13 (0.81, 1.57)
Spinal	0.63 (0.57, 0.69)*	0.57 (0.41, 0.80)*	1.08 (0.91, 1.29)
Regional	0.39 (0.33, 0.47)*	0.66 (0.28, 1.57)*	0.39 (0.29, 0.53)*
Local	0.32 (0.23, 0.43)*	0.33 (0.15, 0.75)*	0.36 (0.14, 0.91)*
Monitored anesthesia care	0.38 (0.34, 0.41)*	0.34 (0.27, 0.44)*	0.44 (0.35, 0.56)*
Other	0.28 (0.17, 0.46)*	0.00 (0.00, *α*)	0.56 (0.19, 1.66)
None	1.09 (0.50, 2.37)	1.08 (0.12, 10.2)	1.44 (0.19, 10.9)
Surgical specialty: vascular	1.69 (1.62, 1.75)*		
Male gender	1.01 (0.98, 1.03)	1.00 (.095, 1.09)	0.90 (0.78, 0.94)*
Ventilator dependent	3.24 (2.87, 3.65)*	3.19 (2.47, 4.13)*	4.22 (2.61, 6.81)*
COPD	1.43 (1.37, 1.50)*	1.60 (1.43, 1.78)*	1.41 (1.23, 1.61)*
Cardiac risk factors	1.27 (1.23, 1.31)*	1.24 (1.12, 1.36)*	1.33 (1.09, 1.63)*
On dialysis	0.91 (0.84, 0.98)*	1.13 (0.95, 1.33)	1.02 (0.89, 1.17)
Steroid use	1.48 (1.39, 1.59)*	1.47 (1.25, 1.72)*	1.07 (0.85, 1.36)
10% weight loss	2.24 (2.07, 2.42)*	2.22 (1.84, 2.67)*	1.47 (1.04, 2.09)*
Sepsis			
None	Reference category	Reference category	Reference category
Sepsis	1.84 (1.72, 1.97)*	1.89 (1.62, 2.19)*	1.65 (1.34, 2.03)*
Septic shock	2.73 (2.40, 3.10)*	3.42 (2.60, 4.49)*	1.98 (1.21, 3.22)*
SIRS	1.63 (1.55, 1.71)*	1.59 (1.40, 1.81)*	1.70 (1.41, 2.04)*
Emergent case	1.57 (1.51, 1.63)*	1.50 (1.35, 1.66)*	1.82 (1.54, 2.14)*
ASA class			
No ASA class assigned	Reference category	Reference category	Reference category
No disturbance	0.42 (0.29, 0.60)*	0.60 (0.17, 2.08	3.39 (0.68, 16.9)
Mild disturbance	1.00 (0.70, 1.42)	0.68 (0.25, 1.85)	1.72 (0.54, 5.42)
Severe disturbance	2.29 (1.62, 3.26)*	1.19 (0.44, 3.24)	2.26 (0.73, 6.95)
Life threatening	4.92 (3.46, 7.00)*	2.51 (0.92, 6.86)	3.99 (1.29, 12.3)*
Moribund	5.85 (3.92, 8.73)*	1.92 (0.64, 5.78)	10.0 (2.37, 42.3)*
Wound classification			
Clean	Reference category	Reference category	Reference category
Clean/contaminated	1.99 (1.93, 2.07)*	1.99 (1.82, 2.16)*	1.32 (1.07, 1.63)*
Contaminated	1.98 (1.88, 2.08)*	2.31 (2.02, 2.63)*	1.31 (1.06, 1.62)*
Dirty/infected	2.38 (2.25, 2.51)*	2.46 (2.15, 2.81)*	1.15 (0.98, 1.35)

Values expressed as odds ratios (95% confidence intervals *x*, *y*). *Significance at *P* < 0.05. COPD: chronic obstructive pulmonary disease, ASA: American Society of Anesthesiologists, SIRS: systemic inflammatory response syndrome, and CI: confidence interval.
